# A Proposed Procedure for Discriminating between Nasal Secretion and Saliva by RT-qPCR

**DOI:** 10.3390/diagnostics10080519

**Published:** 2020-07-26

**Authors:** Tomoko Akutsu, Ken Watanabe

**Affiliations:** National Research Institute of Police Science, 6-3-1 Kashiwanoha, Kashiwa-shi, Chiba 277-0882, Japan; k-watanabe@nrips.go.jp

**Keywords:** nasal secretion, saliva, RT-qPCR, body fluid identification

## Abstract

In forensic casework, nasal secretion can be a good source of DNA. Moreover, saliva can prove useful in cases of sexual assault. However, discriminating between these body fluids is often difficult because of cross-reactivity between them on presumptive and confirmatory tests. Therefore, an RT-qPCR procedure was developed to discriminate between nasal secretion and saliva. Characteristic genes in nasal secretion and/or saliva (*BPIFA1*, *STATH*, *HTN3,* and *PRH2*) were selected as candidates. Discrimination criteria were established based on the expression levels of these markers in various body fluids. In addition, a flowchart was proposed and used to discriminate among nasal secretion, saliva, and other body fluids in various forensic samples. *BPIFA1* was highly expressed in nasal secretion but was also expressed in saliva, semen, and vaginal fluid at trace levels. *STATH* was expressed in nasal secretion and saliva but not in other body fluids. *HTN3* was specifically expressed in most of the saliva samples, as reported previously. Unexpectedly, *PRH2* was expressed in only a few saliva samples. Using the proposed criteria and flowchart, nasal secretion and saliva were successfully discriminated among the various body fluids tested. The developed procedure could be useful in forensic casework.

## 1. Introduction

In criminal casework, various types of biological samples are recovered from crime scenes. These biological samples are analyzed to determine what type of crime may have occurred and whether a particular suspect was involved. Nasal secretion can be a good source of DNA for the purpose of individual identification, but there are few procedures to identify nasal secretion. Further, saliva is one of the main body fluids left at crime scenes, and analysis results can prove useful in various criminal cases, especially sexual assaults. However, presumptive and confirmatory tests for saliva based on α-amylase activity or protein [1,2,3] can show cross-reactivity with other body fluids [4,5,6,7], such as nasal secretion. Therefore, a procedure for discriminating between nasal secretion and saliva could be valuable in criminal casework.

In recent decades, gene expression analysis of mRNA markers characteristic of body fluids has been used for the forensic identification of biological samples [8,9,10,11,12,13]. Those studies reported bactericidal permeability-increasing protein fold-containing family member A1 (BPIFA1) as a nasal secretion marker [14,15]. BPIFA1 is a lipid-binding protein that plays a role in the innate immune responses of the upper airway [16,17]. In addition, *statherin* (*STATH*) and *histatin 3* (*HTN3*) have been widely used as markers characteristic of saliva [8,9,18]. STATH is specifically secreted from the salivary glands, and it plays a role in stabilizing saliva supersaturated with calcium salts by inhibiting the precipitation of calcium phosphate salts and modulating hydroxyapatite crystal formation on the tooth surface [19,20]. HTN3 is also secreted from the salivary glands. It is considered to be a major precursor of the protective proteinaceous structure on tooth surfaces (enamel pellicle) and exhibits antibacterial and antifungal activities [21,22]. However, endpoint detection of these genes might be difficult for discriminating between nasal secretion and saliva because of their insufficient specificity and detectability [14,15].

Therefore, the aim of this study was to develop a more specific procedure for discriminating between nasal secretion and saliva for forensic purposes. First, for the quantitative evaluation of candidate molecules, reverse-transcription quantitative polymerase chain reaction (RT-qPCR) procedures were developed to identify nasal secretion (*BPIFA1* and *STATH*) and saliva (*STATH*, *HTN3,* and proline-urinrich protein HaeIII subfamily 2 [*PRH2*]). PRH2 is a member of the proline-rich protein family that acts as a highly potent inhibitor of calcium phosphate crystal growth [23,24]. These proteins provide a protective and reparative environment for dental enamel, which is important for the integrity of the teeth [25]. Because *PRH2* is specifically expressed in the salivary glands [26], and enzyme-linked immunosorbent assay (ELISA) of PRH2 is specific for saliva [27,28], we selected it as an additional candidate for saliva. Expression levels of candidate genes in various body fluids were determined, and discrimination criteria for nasal secretion and saliva were established based on the quantitative results of multiple markers. In addition, a flowchart was proposed to discriminate among nasal secretion, saliva, and other body fluids in various forensic samples. This is a developmental experiment that involves a qPCR procedure, and therefore, we prepared this report in accordance with the Minimum Information for Publication of Quantitative Real-Time PCR Experiments (MIQE) guidelines [29,30,31].

## 2. Materials and Methods 

### 2.1. Sample Collection

Nasal secretion was collected as stains (*n* = 9) from tissues into which volunteers blew or as fluid (*n* = 1) from the nose using a pipette. Saliva, semen, and urine samples (*n* = 16, 9, and 6, respectively) were collected from volunteers in sterile centrifuge tubes using conventional non-invasive methods. Vaginal fluid stains (*n* = 8) were obtained from premenopausal women by wiping the vaginal wall with a sterile cotton swab (ø = 12 mm). The phase of the menstrual cycle was not restricted and was not asked about for these samples. Blood (*n* = 7) was collected from the brachial vein of volunteers in blood collection tubes containing EDTA as an anticoagulant. All samples were stored at −80 °C until analysis.

### 2.2. RNA Extraction and cDNA Synthesis 

Total RNA extraction, DNase digestion and cDNA synthesis from all the samples were performed as previously reported [9,32]. Total RNA was extracted using an RNeasy Mini Kit (Qiagen, Hilden, Germany) from 1 × 2-cm square pieces of tissue paper, 30 μL of fluid or 5 × 5-mm square pieces of swab head. Possibly contaminated DNA was digested using an RNase-Free DNase Set (Qiagen, Germantown, MD, USA). An aliquot of 5 μL of DNA-free total RNA was added to 10 μL of the reverse transcription mixture of a Primescript RT Reagent Kit (Takara Bio, Otsu, Japan). All procedures were performed according to the manufacturer’s instructions. Successful removal of DNA contaminants was confirmed using no-reverse transcription controls of representative samples for each type of body fluid.

### 2.3. Amplification of Candidate Genes by a qPCR Procedure

An RT-qPCR procedure was developed using the SmartCycler II system (Cepheid, Sunnyvale, CA, USA) with SYBR premix DimerEraser (Takara Bio, Otsu, Japan). An aliquot of 1 μL of prepared cDNA was added to 25 μL of PCR mixture that contained 0.3 μM of primers. Gene accession number, primer sequences, amplicon length, and amplifiable splicing variants for candidate genes are listed in Table 1. Primer pairs for candidate genes except *STATH* were designed using Primer3 (https://www.ncbi.nlm.nih.gov/tools/primer-blast/). Primer sequences for *STATH* were previously reported [18]. *Actin beta* (*ACTB*) was used as a reference gene [33,34] to confirm the successful preparation of cDNAs and normalize the expression levels of candidate genes. Amplification was conducted under the following conditions: initial denaturing at 95 °C for 30 s, followed by 40 cycles at 95 °C for 5 s, 60 °C for 30 s, and 72 °C for 30 s. Next, a melting curve analysis was performed from 95 °C to 60 °C. A no-template control was used in each batch of the PCR mixture as a negative control. The specificity of the amplification was confirmed by melting curve analysis and sequencing. Direct sequencing of amplicons was performed using a BigDye™ Terminator v1.1 Cycle Sequencing Kit and a 3100-Avant Genetic Analyzer (Thermo Fisher Scientific, Waltham, MA, USA).

### 2.4. Validation of the Developed RT-qPCR Procedure and Gene Expression Analysis of Candidate Genes

The cycle quantification (Cq) value was determined to be the crossing point of a primary amplification curve and the default threshold value (fluorescence unit = 30) [35].

Standard curves were drawn using a serial-diluted representative nasal secretion cDNA and salivary gland cDNA (PCR Ready First Strand cDNA, Biochain, Newark, CA, USA) for *BPIFA1* and the other genes, respectively. The linear dynamic range was defined as the range where the correlation coefficient (*r^2^*) of the standard curve was > 0.99. Amplification efficiency (E) was determined from the slope of the standard curve as follows:

E = 10^−1/slope^ − 1 (1)

The cutoff Cq value of each gene was determined as the upper Cq value of the linear range (*r^2^* > 0.99). Cq variation at the lower limit of the standard curve was calculated as a standard deviation to evaluate the repeatability of the developed procedure.

For the evaluation of candidate genes as markers to discriminate between nasal secretion and saliva, the Cq values were determined in various body fluids. For samples with a Cq value of *ACTB* that was below the cutoff, the Cq value was subjected to further analysis. Then, the cutoff Cq value was set to determine the positivity of each marker in each body fluid. The ΔCq value, the Cq value for each target gene normalized relative to that of *ACTB*, was determined to compare the relative expression levels among various body fluids. The ΔCq value was calculated within the linear range of Cq values of both target and reference genes.

These assays were performed in triplicate to evaluate repeatability and in singlicate for other analyses. All procedures performed in this study were in accordance with the Ethical Guidelines for Human Genome/Gene Analysis Research (Ministry of Education, Culture, Sports, Science and Technology; Ministry of Health, Labour and Welfare; and Ministry of Economy, Trade and Industry of Japan) and with the 1964 Helsinki Declaration and its later amendments or comparable ethical standards. All procedures were approved by the Institutional Ethics Committee for Human Genome, Gene Analysis Research of the National Research Institute of Police Science (the corresponding ethical approval code: #31-2(69), approval date: 5 June 2019).

## 3. Results

### 3.1. Assay Performance of the Developed RT-qPCR Procedure

To assess the assay performance of the developed RT-qPCR procedure, the slope, y-intercept, linear dynamic range and *r*^2^ value of the standard curve for each gene were determined and are summarized in Table 2. The standard curve of *BPIFA1* was drawn using a serial dilution of a representative nasal secretion cDNA. Its lower limit of dilution, which showed *r*^2^ values above 0.99, was 0.0156. Similarly, the lower limits of dilution were 1.53 × 10^−9^ for *STATH* and *HTN3* and 3.81 × 10^−7^ for *PRH2* and *ACTB*, in the standard curves drawn using salivary gland cDNA. Then, the amplification efficiency and cutoff Cq value were determined with the corresponding parameters. Amplification efficiencies of all candidates were between 92.6% and 112.6% (Table 2). In addition, the standard deviations of the Cq values calculated at the lower limit of the linear dynamic range were between 0.2 and 0.63 (Table 2). In this procedure, no Cq values were obtained from negative controls.

### 3.2. Expression of Candidate Genes in Various Body Fluids

To evaluate the applicability of the developed procedure for discriminating between nasal secretion and saliva, RT-qPCR analyses for candidate genes were performed in various forensically relevant body fluids. Then, the cutoff Cq value was adopted to determine the positivity of each marker in each body fluid. In these samples, 5 of 6 urine samples showed above the cutoff Cq value for *ACTB* and determined as negative. It might be caused by the smaller amounts of cellular contents of urine. As shown in Table 3, *BPIFA1* was positive in 7 of 10 nasal secretions and in 1 of 9 semen samples. *STATH* was positive in almost all of the nasal secretion and saliva samples but negative in all of the other body fluids analyzed in this study. *HTN3* was specifically positive in saliva samples, consistent with our previous report [9]. However, only 1 saliva sample showed Cq values above the cutoff value in the RT-qPCR analysis for *PRH2*. One synonymous variant (rs1136515, C > T) with high minor allele frequency (C = 0.25 − 0.50) was found in the middle range of the reverse primer of *HTN3* (https://www.ncbi.nlm.nih.gov/snp/rs1136515). At the same time, almost all of our saliva samples showed positive result in *HTN3* (Table 3). Therefore, the effect of this SNP on primer annealing does not seem to be crucial.

**Figure 1:**
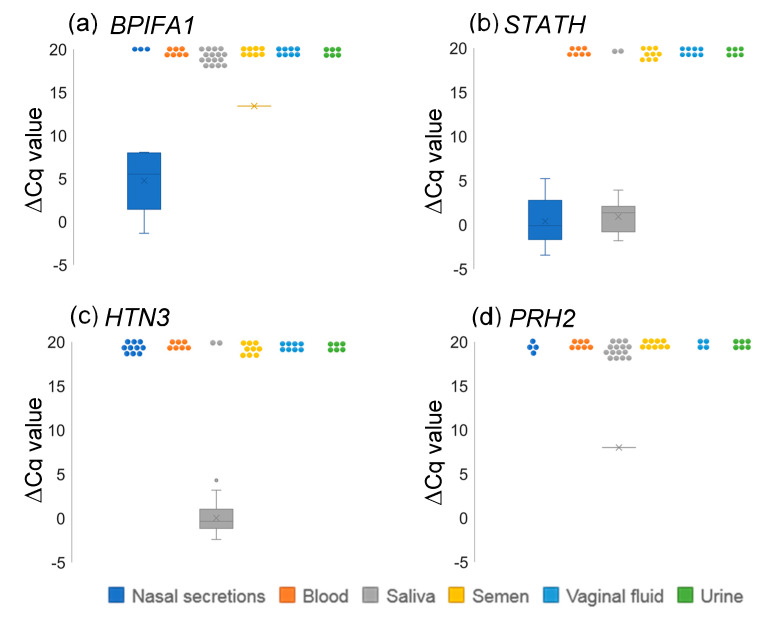
Box plots showing expression levels of *BPIFA1* (**a**), *STATH* (**b**), *HTN3* (**c**), and *PRH2* (**d**) in various body fluids. Each dot at around ΔCq = 20 represents samples that show no amplification or are outside of the linear range.

Expression levels of these genes were also determined in nasal secretion, saliva and other body fluids. As a result, *BPIFA1* was highly but not fully expressed in nasal secretion and was also expressed in semen at trace levels (Figure 1a), although outside of the linear range, *BPIFA1* was also slightly expressed in some samples of saliva, semen and vaginal fluids (Table S1). *STATH* was expressed at comparable levels in the nasal secretion and saliva samples (Figure 1b). As shown in Figure 1c, *HTN3* was specifically expressed in saliva samples with ΔCq values of around 0. In contrast, only 1 saliva sample showed a valid ΔCq value for *PRH2* (Figure 1d), although ΔCq values for other saliva samples are shown for reference only because they were below the cutoff Cq values (Table S1).

### 3.3. Establishing the Discrimination Criteria for Nasal Secretion and Saliva

Because of the lower detectability and expression levels of *PRH2* in saliva, this gene was excluded from further study. *STATH* was specifically positive in nasal secretion and saliva, so the cutoff Cq value could be used as a criterion for discrimination. In addition to *STATH*, the cutoff Cq value of *HTN3* was also used as a criterion. Because *BPIFA1* was highly expressed in nasal secretion but only slightly expressed in saliva, semen and vaginal fluids, a cutoff ΔCq value was proposed to establish the criterion. When the cutoff ΔCq value was set at <10, 7 of 10 nasal secretions were determined to be positive and all the other body fluids including one case of *BPIFA1* detected in semen were determined to be negative. Some saliva samples that were outside the linear range showed ΔCq < 10, and these are reported for reference only (Table S1).

### 3.4. Proposed Flowchart for Discriminating among Nasal Secretions, Saliva, and Other Biological Samples

**Figure 2:**
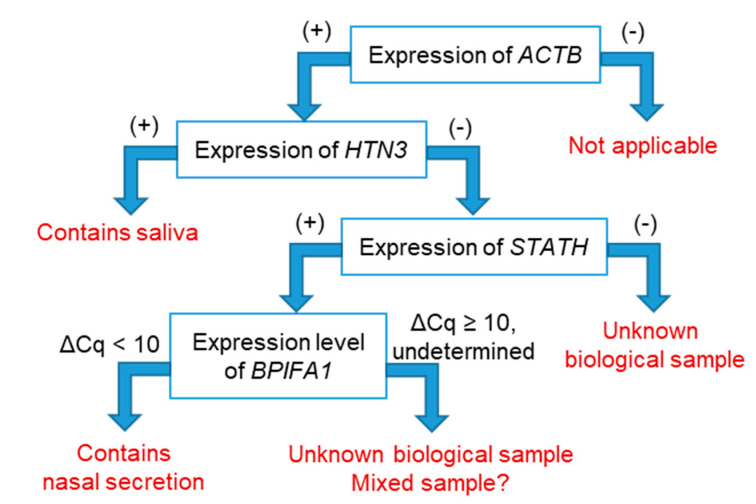
Proposed flowchart for discriminating among nasal secretions, saliva and other body fluids by RT-qPCR. (+), below the cutoff Cq values and determined as positive; (−), above the cutoff Cq values and determined as negative.

To improve the specificity of the discrimination among nasal secretion, saliva and other biological samples, a flowchart was proposed (Figure 2). First, an *ACTB*-positive sample was entered into the flowchart, and if *HTN3* was positive, the sample was considered to be a “saliva-containing” sample. If *HTN3* was negative, the expression of *STATH* was evaluated. If *STATH* was also negative, the sample was considered an “unknown” biological sample. For *STATH*-positive samples, the expression level of *BPIFA1* was evaluated. If the ΔCq value of *BPIFA1* was <10, the sample was considered to be a “nasal secretion-containing” sample. If the ΔCq value of *BPIFA1* was ≥10 or undetermined, the sample was also considered an “unknown” biological sample. As shown in Table 4, nasal secretion and saliva were specifically discriminated among various body fluids analyzed in this study using the proposed flowchart. All the other *ACTB*-positive body fluids were determined to be unknown biological samples; however, 3 of 10 nasal secretions and 2 of 16 saliva samples were also classified as unknown biological samples.

## 4. Discussion

RNA profiling has at least one clear advantage over conventional methods in that different types of body fluids can be analyzed by a unified procedure. In addition, several markers can be detected simultaneously by multiplex RT-PCR [8,36,37]. For these analyses, genes specifically and highly expressed in the targeted body fluid tend to be the best markers. However, such specific genes have not been reported for nasal secretion to date. Saliva is also difficult to identify precisely, because various markers for saliva are detected in other body fluids such as nasal secretion [4,5,6,7,27,28].

Accordingly, in this study, we attempted to develop a specific procedure for discriminating between nasal secretion and saliva for forensic purposes. RT-qPCR procedures were successfully developed for the expression analysis of *BPIFA1*, *STATH,* and *HTN3* as markers of nasal secretion and/or saliva. Then, discrimination criteria were established for the positive detection of these genes. Moreover, a discrimination flowchart was proposed to improve the specificity for nasal secretion and saliva. As a result, these body fluids were successfully discriminated from among various body fluids.

Unfortunately, only a few saliva samples showed positive results in the RT-qPCR analysis for *PRH2*, although PRH1/2, an alias of PRH2, was detected in most of the saliva samples by ELISA as a saliva-specific protein marker [27,28]. Although the cause of this discrepancy is unclear, mRNA expression is not necessarily comparable to protein expression. On targeted RNA sequencing for forensic body fluid identification, there were fewer read counts of *PRH2* than of other saliva markers, but it was detected only in saliva [38]. Our result supports this previous study, and gene expression analysis of *PRH2* seems to be difficult to apply to forensic identification of saliva.

Because the sample size and conditions were limited in this preliminary study, discrimination should be validated in additional samples which were collected from volunteers of all ages. For example, although all of 9 semen and 8 vaginal fluid samples analyzed in this study were negative for *STATH*, it has been reported to be expressed at trace levels in such fluids [11,33,39]. The cutoff ΔCq value of *STATH* could be effective in avoiding misclassification of semen and vaginal samples as “saliva-containing”. In addition, we will perform practical evaluation for the forensic application of the proposed procedure with artificially degraded samples, mixed samples, and mock casework samples. Furthermore, because all the other body fluids were classified as “unknown biological samples” using the flowchart proposed in this study, we are currently evaluating additional markers for the determination of other body fluids.

## 5. Conclusions

Nasal secretion and saliva were successfully discriminated from among various body fluids by a combination of RT-qPCR analysis of *BPIFA1*, *STATH*, and *HTN3* genes and use of the proposed flowchart.

## Figures and Tables

**Table 1:** Primers for nasal secretion and saliva characteristic target genes and the reference gene.

Gene	Accession No.	Forward Primer Sequence (5′–3′)	Amplicon Length (bp)	Splicing Variant	Reference
		Reverse Primer Sequence (5′–3′)			
*BPIFA1*	NM_016583.3	CCTTGGTGACTGCACCCATT	161	1–3	This study
		CCTCATTGACCAGAGGGCAC			
*STATH*	NM_003154.2	TTTGCCTTCATCTTGGCTCT	93	1	[18]
		CCCATAACCGAATCTTCCAA			
*HTN3*	NM_000200.3	CATGACTGGAGCTGATTCACA	135	-	This study
		ATGCCCCGTGATTACTGAAGA			
*PRH2*	NM_001110213.1	GGGCAGTCTCCTCAGTAATCTA	166	-	This study
		CCCAAACACTCAGAAGGAGATG			
*ACTB*	NM_001101.5	TGGCACCCAGCACAATGAA	186	-	Takara Bio ^1^
		CTAAGTCATAGTCCGCCTAGAAGCA			

^1^ Designed by Perfect Real Time Support System (Takara Bio Inc., Otsu, Japan).

**Table 2:** Summary of the performance of the developed reverse-transcription quantitative polymerase chain reaction (RT-qPCR) procedure.

Gene	Slope	Y-Intercept	Lower Limit of Linear Dynamic Range	*r* ^2^	Amplification Efficiency	Cutoff Cq Value	Cq Variation ^3^
*BPIFA1* ^1^	−3.51	29.17	0.0156	0.996	92.6%	35.68	0.63
*STATH* ^2^	−3.20	9.31	1.53 × 10^−9^	0.994	105.3%	36.97	0.21
*HTN3* ^2^	−3.05	8.99	1.53 × 10^−9^	1.000	112.6%	35.84	0.54
*PRH2* ^2^	−3.29	15.66	3.81 × 10^−7^	0.997	101.4%	36.39	0.57
*ACTB* ^2^	−3.19	15.61	3.81 × 10^−7^	0.996	105.8%	35.54	0.20

^1^ The standard curve was plotted using cDNA prepared from a representative sample of nasal secretion. ^2^ The standard curve was plotted using purchased salivary gland cDNA. ^3^ Standard deviation of Cq value at the lower limit of the linear dynamic range.

**Table 3:** Detectability of candidate genes for nasal secretion and saliva discrimination in various body fluids.

Body Fluid	Number of Tested Samples	Number of Positive Samples ^1^				
		*ACTB*	*BPIFA1*	*STATH*	*HTN3*	*PRH2*
Nasal secretion	10	10	7	10	0	0
Saliva	16	16	0	14	14	1
Blood	7	7	0	0	0	0
Semen	9	9	1	0	0	0
Vaginal fluid	8	8	0	0	0	0
Urine	6	1	0	0	0	0

^1^ Cq values above the cutoff value were regarded as positive.

**Table 4:** Results of discrimination according to the proposed flowchart.

Target Fluid	Sensitivity	Specificity
Nasal secretion	70%	100%
Saliva	87.5%	100%
Others ^1^	83.3%	80.8%

^1^ Evaluated for samples classified as unknown biological sample.

## Supplementary Materials

The following are available online at https://www.mdpi.com/2075-4418/10/8/519/s1. Table S1. The Cq and ΔCq values of target genes and the reference gene in various forensically relevant body fluids.
