# Supplementary material for: A Proposed Procedure for Discriminating between Nasal Secretion and Saliva by RT-qPCR
Source: Diagnostics (Basel). 2020 Jul 26;10(8):519. doi: 10.3390/diagnostics10080519 (PMC7460356; doi:10.3390/diagnostics10080519)
Supplement: Supplementary file 1 [file diagnostics-10-00519-s001.pdf]

Table S1. The Cq and ΔCq values of target genes and the reference gene in various forensically relevant body fluids.

	Cq values					ΔCq (Target gene – reference gene) values			
Body fluids	*ACTB*	*BPIFA1*	*STATH*	*HTN3*	*PRH2*	*BPIFA1*	*STATH*	*HTN3*	*PRH2*
Nasal secretion	32.32	N.D.[1]	30.55	N.D.	N.D.	- [2]	−1.77	-	-
	28.43	34.97	28.64	N.D.	N.D.	6.54	0.21	-	-
	35.21	33.87	31.77	N.D.	N.D.	−1.34	−3.44	-	-
	35.34	N.D.	33.71	N.D.	N.D.	-	−1.63	-	-
	27.45	32.62	29.82	N.D.	N.D.	5.17	2.37	-	-
	29.55	35.07	29.15	N.D.	N.D.	5.52	−0.4	-	-
	31.38	36.49 [3]	31.86	N.D.	N.D.	5.11	0.48	-	-
	27.35	35.34	31.30	N.D.	N.D.	7.99	3.95	-	-
	30.47	31.91	29.45	N.D.	N.D.	1.44	−1.02	-	-
	22.85	30.9	28.08	N.D.	N.D.	8.05	5.23	-	-
Saliva	31.39	N.D.	29.59	29.07	37.10	-	−1.8	−2.32	5.71
	34.47	N.D.	36.57	33.66	N.D.	-	2.1	−0.81	-
	28.37	N.D.	30.16	27.93	36.37	-	1.79	−0.44	8.00
	32.25	N.D.	34.34	31.78	N.D.	-	2.09	-0.47	-
	35.41	N.D.	34.58	34.84	N.D.	-	−0.83	−0.57	-
	32.53	N.D.	33.08	32.75	N.D.	-	0.55	0.22	-
	31.31	N.D.	32.55	31.35	N.D.	-	1.24	0.04	-
	32.10	N.D.	35.49	35.29	N.D.	-	3.39	3.19	-
	33.03	N.D.	31.93	32.80	N.D.	-	−1.1	−0.23	-
	28.29	N.D.	29.84	29.30	N.D.	-	1.55	1.01	-
	34.66	N.D.	39.50	N.D.	N.D.	-	4.84	-	-
	28.12	N.D.	32.05	32.43	36.67	-	3.93	4.31	8.55
	35.31	N.D.	39.64	39.03	N.D.	-	4.33	3.72	-
	31.30	38.3	30.52	29.19	38.49	7.00	−0.78	−2.11	7.19
	30.70	N.D.	30.10	28.30	N.D.	-	−0.60	−2.40	-
	28.99	37.56	30.49	30.09	39.20	8.57	1.5	1.10	10.21
Blood	21.69	N.D.	N.D.	N.D.	N.D.	-	-	-	-
	22.05	N.D.	N.D.	N.D.	N.D.	-	-	-	-
	22.95	N.D.	N.D.	N.D.	N.D.	-	-	-	-
	22.08	N.D.	N.D.	N.D.	N.D.	-	-	-	-
	23.15	N.D.	N.D.	N.D.	N.D.	-	-	-	-
	22.92	N.D.	N.D.	N.D.	N.D.	-	-	-	-

	23.15	N.D.	N.D.	N.D.	N.D.	-	-	-	-
Semen	23.91	36.67	N.D.	N.D.	N.D.	12.76	-	-	-
	25.88	N.D.	N.D.	N.D.	N.D.	-	-	-	-
	26.26	N.D.	N.D.	N.D.	N.D.	-	-	-	-
	25.95	N.D.	N.D.	N.D.	N.D.	-	-	-	-
	22.07	35.49	N.D.	N.D.	N.D.	13.42	-	-	-
	23.59	35.8	N.D.	N.D.	N.D.	12.21	-	-	-
	23.12	36.75	N.D.	N.D.	N.D.	13.63	-	-	-
	24.86	N.D.	N.D.	N.D.	N.D.	-	-	-	-
	23.17	35.84	N.D.	N.D.	N.D.	12.67	-	-	-
Vaginal fluid	26.09	N.D.	N.D.	N.D.	N.D.	-	-	-	-
	26.26	N.D.	N.D.	N.D.	N.D.	-	-	-	-
	23.83	36.19	N.D.	N.D.	N.D.	12.36	-	-	-
	25.93	N.D.	N.D.	N.D.	N.D.	-	-	-	-
	25.52	N.D.	N.D.	N.D.	N.D.	-	-	-	-
	23.88	N.D.	N.D.	N.D.	N.D.	-	-	-	-
	23.14	N.D.	N.D.	N.D.	N.D.	-	-	-	-
	25.13	N.D.	N.D.	N.D.	N.D.	-	-	-	-
Urine	37.89	N.D.	N.D.	N.D.	N.D.	-	-	-	-
	33.72	N.D.	N.D.	N.D.	N.D.	-	-	-	-
	38.20	N.D.	N.D.	N.D.	N.D.	-	-	-	-
	37.39	N.D.	N.D.	N.D.	N.D.	-	-	-	-
	37.72	N.D.	N.D.	N.D.	N.D.	-	-	-	-
	37.49	N.D.	N.D.	N.D.	N.D.	-	-	-	-

[1] N.D., Not detected; [2], Not calculated because the Cq value of target gene was not determined; [3] The Cq and ΔCq values highlighted in red font were shown for reference only because they were above the cutoff Cq values (Cq(*BPIFA1*) > 35.68, Cq(*STATH*) > 36.97, Cq(*HTN3*) > 35.84, Cq(*PRH2*) > 36.39 and Cq(*ACTB*) > 35.54).
